# Harmaline induces apoptosis and inhibits migration in A2780 ovarian cancer cells in vitro

**DOI:** 10.14814/phy2.15984

**Published:** 2024-03-26

**Authors:** Seyed Ali Shariat Razavi, Masoumeh Taghdisi Khaboushan, Raha Jafari, Arshia Shahini, Gordon A. Ferns, Afsane Bahrami

**Affiliations:** ^1^ Department of Neuroscience, Faculty of Medicine Mashhad University of Medical Sciences Mashhad Iran; ^2^ Student Research Committee Islamic Azad University Mashhad Branch Mashhad Iran; ^3^ Department of Medicine, Mashhad Medical Sciences Branch Islamic Azad University Mashhad Iran; ^4^ Department of Laboratory Sciences, School of Allied Medical Sciences Arak University of Medical Sciences Arak Iran; ^5^ Division of Medical Education Brighton and Sussex Medical School Falmer, Brighton, Sussex UK; ^6^ Clinical Research Development Unit, Imam Reza Hospital, Faculty of Medicine Mashhad University of Medical Sciences Mashhad Iran; ^7^ Clinical Research Development Unit of Akbar Hospital, Faculty of Medicine Mashhad University of Medical Sciences Mashhad Iran

**Keywords:** apoptosis, migration, *Peganum harmala*

## Abstract

Ovarian cancer is one of the most prevalent malignancies in women. Harmaline is reported to have powerful anticancer properties. We aimed to investigate the apoptotic and antimetastatic properties of harmaline in A2780 ovarian cancer cells. Cell viability, apoptosis, migration, and invasion were investigated in cells treated with harmaline. Reactive oxygen species (ROS) production, mRNA expression of apoptosis‐associated genes, MMP‐2, and MMP‐9 were measured. Harmaline attenuated the viability of A2780 ovarian cancer cells in a dose‐ and time‐dependent way. Furthermore, compared to NIH/3T3 mouse normal cell line (IC50 = 417 μM), the malignant A2080 cells were more sensitive to harmaline (IC50 = 300 μM after 24 h). Harmaline increased the production of ROS, raised the mRNA expression of p53 and the Bax/Bcl2 ratio. Harmaline also increased the proportion of cells in the late apoptotic and necrotic phases. MMP‐2 and MMP‐9's mRNA expression, gelatinase activity, and migration of A2780 cells also decreased by harmaline. These findings suggest that harmaline may have the potential to be a therapeutic drug for ovarian cancer by triggering apoptosis and suppressing invasion and migration.

## 1 INTRODUCTION

Ovarian cancer (OC) is the eighth most prevalent cancer in women, and is among the five most common types of gynecologic cancer with a high mortality rate (Haslehurst et al., 2012). About 90% of ovarian tumor cases are epithelial OC, and approximately 60% of these cases are further categorized as serous carcinoma with extremely diverse features (Xu et al., 2017). Because of the highly invasive nature of OC cells and the lack of a reliable method of early detection, a high percentage of patients suffer from distant metastases. In the majority of OC cases, surgery is the recommended treatment option, and chemotherapy is usually necessary to remove the lesions. Even though platinum‐taxane doublet‐based chemotherapy (PT‐DC) has been the first‐line therapy for the past decades, chemo‐resistance has occurred in more than 70% of patients (Jayson et al., 2014; Yeung et al., 2015). Therefore, to improve treatment results, it is vital to define novel therapeutic approaches related to OC.

There is increasing evidence that some natural products may provide a novel source of therapeutic approaches. Among the many widely used natural products, the anticancer potential has been deemed critical. Natural products have contained approximately 77% of all small molecules used in the treatment of different neoplasms (Sharifi‐Rad et al., 2019). Some of these compounds, such as mitomycin C and actinomycin D from bacteria, paclitaxel, etoposide, vincristine, and irinotecan from plants, have demonstrated notable antitumor activity on different cancer cells (Huang et al., 2021). A class of natural and synthetic indole alkaloids known as “β‐carboline alkaloids” has a tricyclic pyrido[3,4‐b] indole ring moiety (Cao et al., 2007). Several medicinal plants, including *Passiflora incarnata*, *Tribulus terrestris*, and Grewia bicolor contain indole alkaloids (Nikam et al., 2013). Harmaline (7‐methoxy‐3,4‐dihydro‐β‐carboline) is a pharmacological derivative of β‐carboline alkaloids which was first extracted from the seeds of *Peganum harmala* (Syrian rue) (Asgarpanah & Ramezanloo, 2012). The primary mechanism of action of harmaline and related ingredients is that they inhibit monoamine oxidase (MAO). They inhibit the metabolism of monoamine compounds such as norepinephrine and serotonin, which in turn stimulated the central nervous system (Handforth, 2012).

Harmaline has been proposed to have numerous therapeutic uses, such as relief from psoriasis, immunosuppression, antipruritic effects, analgesia, reduction of inflammation, protection from radiation, and anticancer activities (Nasehi et al., 2016). In particular, the current study focused on the potential anticancer effects of harmaline, which were originally investigated in the 1970s (Nasehi et al., 2016). The ability of harmaline to modulate the cellular and signal transductions implicated in oxidative balance, DNA integrity maintenance, cell cycle progression, and apoptosis regulation appears to be the basis for the antitumor effects of this alkaloid (Bhattacharjee et al., 2018; Rashidi et al., 2022). It has been reported that harmaline can suppress the proliferative activity of several cancerous cells, including esophageal squamous cell carcinoma (ESCC) (Zhang et al., 2021), myeloma (Boeira et al., 2001), as well as lung (Roy et al., 2020), breast (Rashidi et al., 2022), liver (Xu et al., 2018), and gastric cancers (Wang et al., 2015). Furthermore, in terms of harmaline's anticancer effects, this alkaloid can be used as an herbal adjuvant alongside chemotherapeutic drugs to assess the drug's efficacy on cancerous cells (Rashidi et al., 2022). Hence, in this investigation, we have explored the anticancer properties of harmaline on A2780 OC cells. Furthermore, the apoptosis‐inducing effects of harmaline were examined, as were the mechanisms of suppression of migration and invasion in this type of tumor cell.

## 2 METHODS AND MATERIALS

### 2.1 Chemical reagents

Trypsin–EDTA, fetal bovine serum (FBS), and RPMI1640 medium were purchased from Gibco (Grand Island, NY, USA). The Abcam company provided the dichloro‐dihydro‐fluorescein diacetate (DCFDA)/H2DCFDA‐cellular reactive oxygen species (ROS) detection assay kit (#ab113851, United Kingdom). An annexin V‐FITC assay kit was purchased from Cayman Chemical (#Item No. 600300, Michigan, MI, USA). MTT powder was purchased from Sigma‐Aldrich (St. Louis, MO, USA). Golexir Pars Co. (Mashhad, Iran) provided the harmaline. Harmaline was dissolved in DMSO (<0.01%), and all tests included a control group with DMSO at a specified concentration. In addition, according to the company report, the purity reported to be ≥95.0% (HPLC) (http://www.golexir.com/harmaline/). In addition, just a single batch was used for all the experiment. All additional compounds, unless otherwise specified, were purchased from Sigma‐Aldrich and Merck (Darmstadt, Germany).

### 2.2 Cell cultures

The human ovarian carcinoma cell line A2780 and the NIH/3T3 cell line were both purchased from the Iran Pasteur Institute in Tehran, Iran. Both of these cell lines were cultured in RPMI1640 medium that was supplemented with 10% fetal bovine serum, streptomycin (100 g/mL), and penicillin100 (U/mL). The cells were grown in an environment that was humidified and kept at 37°C in 5% CO_2_.

### 2.3 Cell viability assay

The cellular toxicity induced by harmaline was assessed using the MTT cell viability method. In 96‐well plates, 1.2 × 10^5^ A2780 and NIH/3T3 cells were cultured in RPMI 1640 with FBS and kept at 37°C in 5% CO_2_. After 24 h, the culture medium was removed, and each well received a medium containing different concentrations of harmaline (0–2000 μM). The cells were tested for viability using the MTT assay (#CAS No. 298‐93‐1) after 24 and 48 h of incubation at 37°C according to standard procedure.

### 2.4 Quantitative reverse transcription‐ (qRT‐) PCR

Total RNA was isolated using a Favorgen RNA extraction kit (#FKBRK 001, Favorgen Biotech) used in accordance with the manufacturer's instructions from the harmaline‐treated A2780 cells (150 and 300 μM). The purity and concentration of the extracted RNA were assessed using UV spectrophotometry (NanoDrop 1000TM, USA) and agarose gel electrophoresis. An easy cDNA synthesis kit (#A101161, Parstous, Iran) was used to reverse transcribe total RNA into cDNA. RealQ Plus 2X‐MasterMix Green without RoxTM (#A323402, Amplicon, Denmark) was then used for qRT‐PCR (Roche, USA), with specific primers for the genes GAPDH, Bax, Bcl‐2, P53, MMP‐2, and MMP‐9, listed in Table 1 (Metabion, Germany). The Livak technique was used to evaluate the relative expression of genes.

**TABLE 1:** The sequence of primers.

Gene symbol	Gene name	Primers (5′ → 3′)	Accession number
Bax	Bcl‐2‐associated X protein	Forward: TGACGGCAACTTCAACTGGG Reverse: CTTCAGTGACTCGGCCAGGG	NM_001291428.2
Bcl‐2	B‐cell lymphoma 2	Forward: GTCATGTGTGTGGAGAGCGTC Reverse: CCGTACAGTTCCACAAAGGCATC	NM_000633.3
P53	Tumor suppressor protein	Forward: ACACGCTTCCCTGGATTGG Reverse: CTAGGATCTGACTGCGGCTC	NM_000546.6
MMP‐2	Matrix metalloproteinase‐2	Forward: GATACCCCTTTGACGGTAAGGA Reverse: CCTTCTCCCAAGGTCCATAGC	NM_001302510.2
MMP‐9	Matrix metalloproteinase‐9	Forward: AGACCTGGGCAGATTCCAAAC Reverse: CGGCAAGTCTTCCGAGTAGT	NM_004994.3
GAPDH	Glyceraldehyde‐3‐phosphate dehydrogenase	Forward: TCAAGATCATCAGCAATGCCTCC Reverse: GCCATCACGCCACAGTTTC	NM_001357943.2

### 2.5 Annexin V‐FITC/PI assay

The annexin V/PI assay was used to measure the rate of cell apoptosis and necrosis. Cells (3 × 10^5^) cells were seeded in a 12‐well and were incubated for 24 h, then exposed to harmaline at a dose of 150 and 300 μM for 24 h. Following cell harvesting, the treated cells were labeled with annexin V‐FITC/PI double‐staining kit. The stained cells were then examined using a BD FACSCalibur flow cytometer (Becton Dickinson, USA). The outcomes were examined with the aid of the program FlowJo V10.

### 2.6 ROS activity evaluation

To assess ROS production in the A2780 cells treated with harmaline, we used a 2′,7′‐dichlorodihydrofluorescein diacetate (DCFH2‐DA, #ab113851) assay kit. After 24 h of 25 × 10^3^ A2780 cells adhesion, cells were incubated with DCFH2‐DA for 45 min while being protected from light. The stained cells were then washed, subjected to harmaline treatments (150 and 300 μM), and given tert‐butyl hydrogen peroxide (TBHP) as a positive control. The effect of co‐treatment with 5 Mm concentration of *N*‐acetyl‐l‐cysteine (NAC) on harmaline‐treated cells was also evaluated. A Victor X5 Multiplable plate reader was used to quantify the fluorescence intensity using a set of filters with 485 excitations and 535 emissions. In addition, the viability of cells was examined in all treated groups using MTT cell viability method. Triplicates of each treatment were carried out.

### 2.7 Scratch wound‐healing method

To examine the migratory potential of harmaline‐treated A2780 cells, a scratch assay was used. In a 6‐well plate, 7 × 10^5^ A2780 cells were seeded. A sterile 100‐μL pipette tip was then used to make a scratch in the cell monolayer, and after that, PBS was used to rinse away any debris or floating cells. The next step was to give harmaline (37.5 and 75 μM) to the cells. Using a Zeiss Axiovert 200 microscope, wound closure was observed and captured at 2 and 48 h (Zeiss, Germany).

### 2.8 Gelatin‐based zymography

Gelatine zymography was used to evaluate the gelatinolytic activities and secretion of two matrix metalloproteinase (MMP‐2 and ‐9) members. In a brief procedure, harmaline (37.5 and 75 μM) was applied to the A2780 cells for 24 h. Following centrifugation of the culture media, 50 μg of total protein was electrophoresed at 12% concentration on an SDS polyacrylamide gel with 0.1 mg/mL gelatine as the substrate. To remove SDS, the gel was washed three times with 2% Triton X‐100 for 60 min. To allow the gelatine to digest, the gel was then incubated for 48 h in an incubation buffer containing 2.5% triton X‐100, 50 mM tris pH 7.4, 5 mM CaCl_2_, and 1 μM ZnCl_2_. The gel was then stained for 60 min with 0.5% coomassie brilliant blue R‐250 and then washed with 25% ethanol plus 10% acetic acid in dH_2_O to reveal the bands. Image J 1.52a software was used to analyze the pictures (NIH, USA).

### 2.9 Matrigel invasion assay

To assess the effectiveness of harmaline in preventing cell invasion, we conducted an experiment using an ECMatrix collagen‐based cell invasion assay (#ECM551, Merck Millipore). A2780 cells were cultured overnight in a medium without fetal bovine serum (FBS), then harvested and suspended in the same FBS‐free medium. The lower part of the chamber contained a substance called DMEM supplemented with 10% FBS, which attracts the cells. However, a chamber without FBS was used as negative control. Harmaline was added to the cell suspension at concentrations of 37.5 and 75 μM, and the cells were incubated for 24 h. A2780 cells invaded and passed through a layer of polymerized collagen, attaching to the bottom of a polycarbonate membrane. After removing the non‐migratory cell layer with a cotton‐tipped swab, the wells were stained with CyQuant GR® dye at room temperature for 20 min. The stained insert was then transferred to a clean well containing extraction buffer for 15 min at room temperature, allowing the dye to be extracted from the underside. The insert was removed from the well, and 100 μL of the dye mixture was transferred to a 96‐well microtiter plate for colorimetric measurement at an optical density of 560 nm. Each treatment was performed in triplicate.

### 2.10 Toxicity studies

Acute toxicity, hepatotoxicity, cytotoxicity, carcinogenicity, mutagenicity, immunotoxicity, adverse outcome pathways (Tox21), and toxicity targets were all predicted using the online web‐based server ProTox‐II.

### 2.11 Statistical analysis

GraphPad Prism® 8.2.1 software was recruited to analyze the data by a one‐way analysis of variance (ANOVA) and Dunnett's post hoc tests. Statistical significance was determined to exist at a *p*‐value of 0.05 or lower. The format for the data presentation was mean and standard deviation.

## 3 RESULTS

### 3.1 Harmaline reduces viability of A2780 cells

**FIGURE 1:**
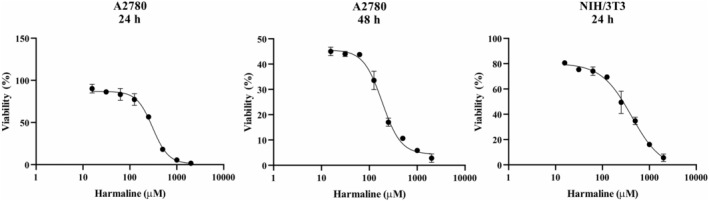
MTT assay for determining cell viability. MTT assay was performed on both A2780 and NIH/3T3 cells to determine cell viability. Cells were treated with a chemical compound called harmaline at different concentrations for 24 and 48 h. The degree of tetrazolium reduction observed in each cell line would be an indicator of the number of viable cells in the culture.

To assess the impact of harmaline on cell viability, MTT assays were conducted on A2780 cells. The MTT assay demonstrated that the viability of A2780 cells was inhibited in a time‐dependent manner. After 24 h of exposure, the IC50 values were approximately 300 μM, and after 48 h of treatment, they were about 185 μM. In order to examine the potentially harmful concentration of harmaline in normal cells, the NIH/3T3 cell line was subjected to different concentrations of harmaline ranging from 0 to 2000 μM over a period of 24 h. As shown in Figure 1, the IC50 value for harmaline in normal NIH/3T3 cells was 417 μM after a treatment period of 24 h. This indicates that malignant cells are more sensitive to harmaline versus normal cells (Figure 1).

### 3.2 Harmaline increases apoptosis in A2780 cells

**FIGURE 2:**
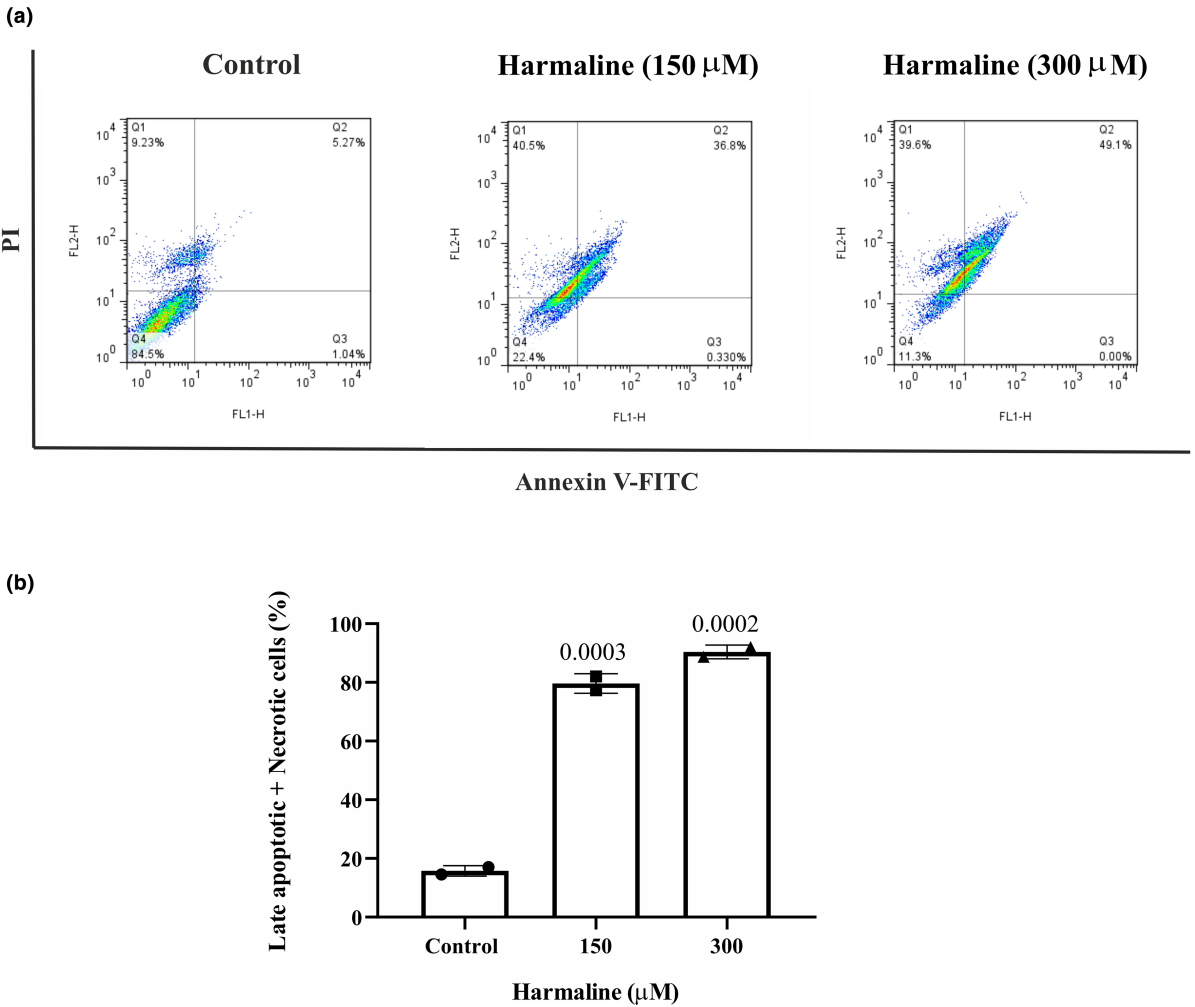
(a) Harmaline induced apoptosis in ovarian cancer cells. The apoptosis of A2780 cells was measured after 24 h harmaline treatment (150 and 300 μM) by flow cytometry. Live cells, early apoptotic cells, late apoptotic cells, and necrotic cells are each represented by Q4 to Q1 in the diagram. (b) The late apoptosis and necrosis were significantly increased dose dependently. The individual data points and significant *p*‐values are shown in each column, and the data are represented as mean ± SD.

**FIGURE 3:**
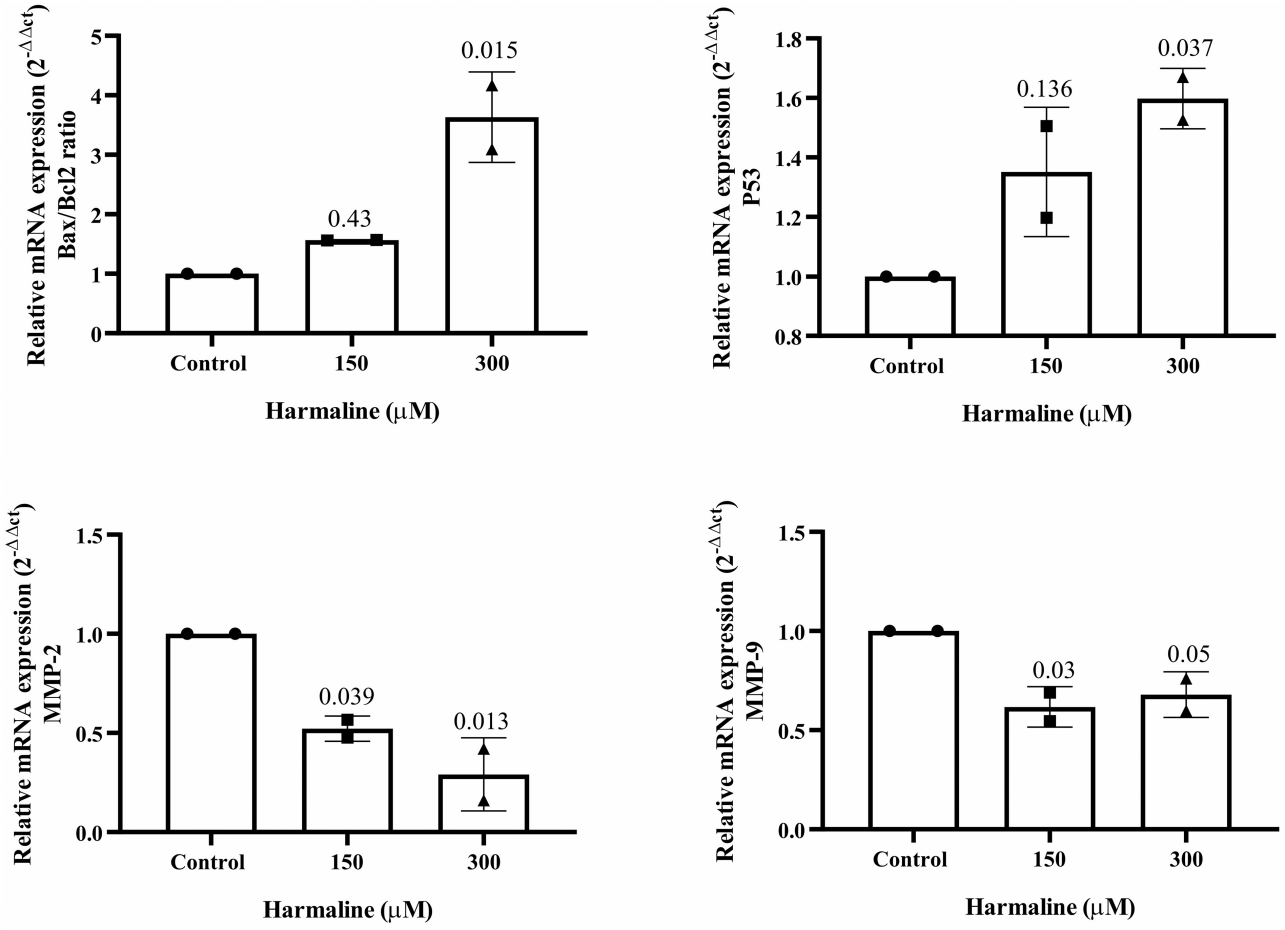
Harmaline induced the expression of Bax and p53 and reduced the expression of MMP2 and MMP9 at the transcription level of ovarian cancer cells. A2780 malignant ovarian cancer cells were individually treated with 150 and 300 μM of harmaline for 24 h, and then the cells were harvested for RT‐PCR to quantify the gene expression of the Bax/Bcl‐2 ratio, p53, MMP‐2, and MMP‐9. The results are presented as means ± standard deviation. The individual data points and significant *p*‐values are shown in each column.

The apoptosis of harmaline‐treated A2780 cells was analyzed by flow cytometry. Treating cells with 150 μM of harmaline caused a significant increase in the rate of apoptotic cells in both the late apoptotic phase (36.8%) and the necrotic phase (40.5%). When tumor cells were treated with 300 μM, the percentages of late apoptotic cells and necrotic cells were, respectively, 49.1% and 39.6%. In the control group, only 5.27% and 9.23% of the cells had progressed to the late and necrotic phases, respectively (Figure 2a). In contrast, early apoptosis was not significantly different between the control (1.4%), 150 μM of harmaline (0.33%), and 300 μM of harmaline (0%). Overall, these results showed that harmaline at both concentrations (150 and 300 μM) significantly increase the late apoptosis and necrosis of A2780 ovarian cells (Figure 2b). In addition, we conducted an assessment of mRNA gene expression related to apoptosis, focusing on key genes such as p53, Bax, and Bcl‐2, with the aim of better understanding the antitumor potential of harmaline. As depicted in Figure 3 our findings revealed a notable increase in the transcription levels of p53 and the Bax/Bcl‐2 ratio within the groups exposed to harmaline, compared to the control group.

### 3.3 Harmaline induced the production of ROS in A2780 cells

**FIGURE 4:**
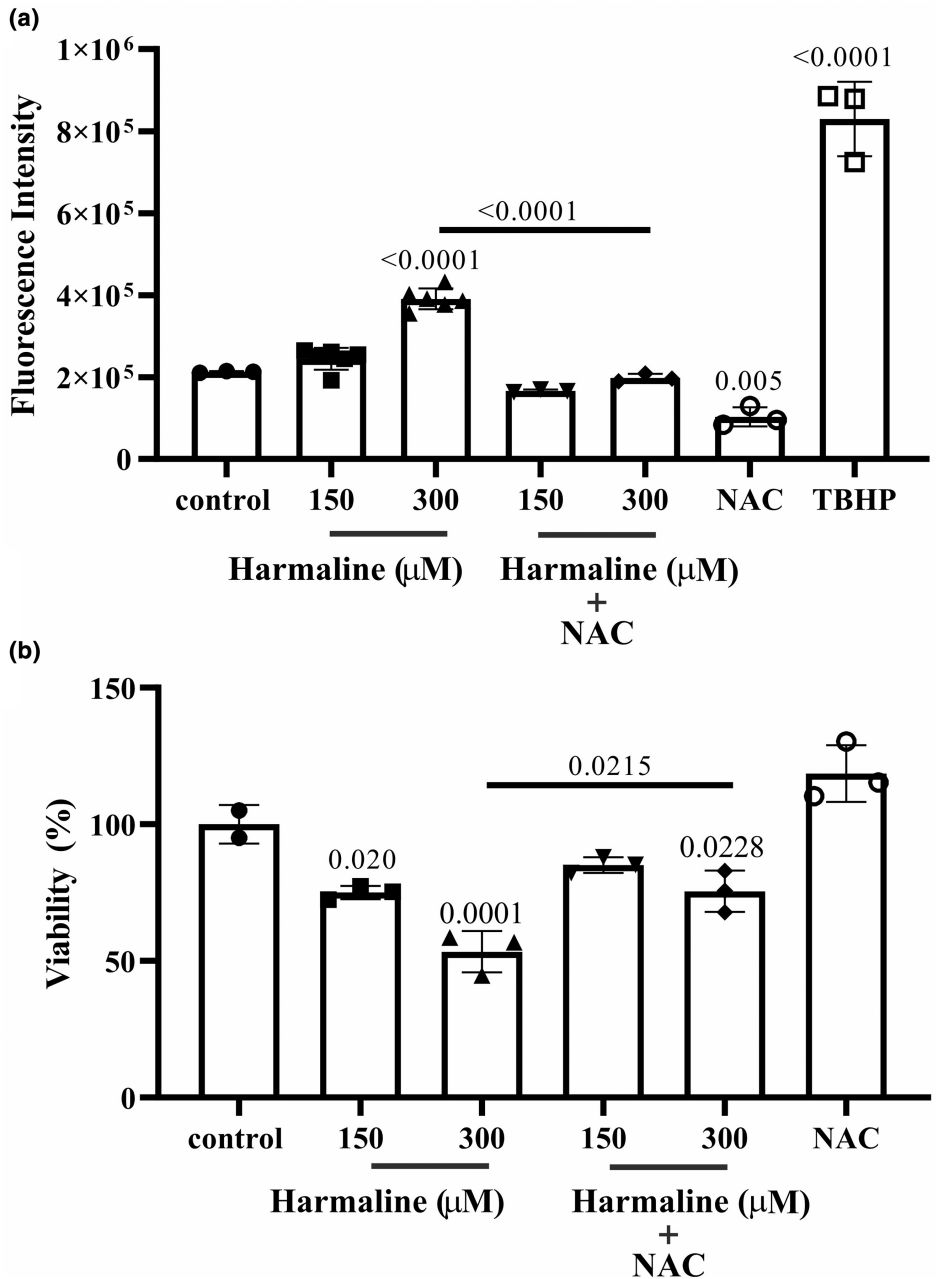
Harmaline induced ROS production in A2780 cells in vitro. (a) After a 24‐h period, we examined the impact of harmaline on the generation of reactive oxygen species (ROS) in A2780 cells using DCFDA as a fluorescent dye. The production of ROS in A2780 cells showed a significant increase when treated with 300 μM of harmaline. However, when *N*‐acetyl cysteine (NAC) was administered along with harmaline, there was a notable inhibition of harmaline‐induced ROS production. As a positive control, tert‐butyl hydroperoxide (TBHP) was introduced to A2780 cells, which resulted in a significantly higher ROS production compared to the control. (b) Combining *N*‐acetyl cysteine (NAC) with harmaline at concentrations of 300 μM resulted in enhanced viability of A2780 cells 24 h after treatment, as compared to each group individually with the same concentrations. The results are expressed as means ± standard deviation. The individual data points and significant *p*‐values are shown in each column.

To investigate the role of ROS in harmaline‐induced cell death, the levels of ROS were measured using fluorimetry. As shown in Figure 4a, treatment of A2780 cells with harmaline for 24 h resulted in an increase in ROS levels and oxidative damage to the cells. However, the addition of NAC, a ROS inhibitor, effectively reduced the elevation of ROS caused by harmaline. As a positive control, TBHP, an inducer of ROS generation, significantly increased ROS levels compared to the control group (Figure 4a). Additionally, Figure 4b demonstrates that NAC reversed the decrease in cell viability observed in harmaline‐treated cells at 24 h. These results suggest that ROS plays a significant role in harmaline‐induced cytotoxicity in A2780 cells.

### 3.4 Harmaline inhibited the cell motility in A2780 cells

**FIGURE 5:**
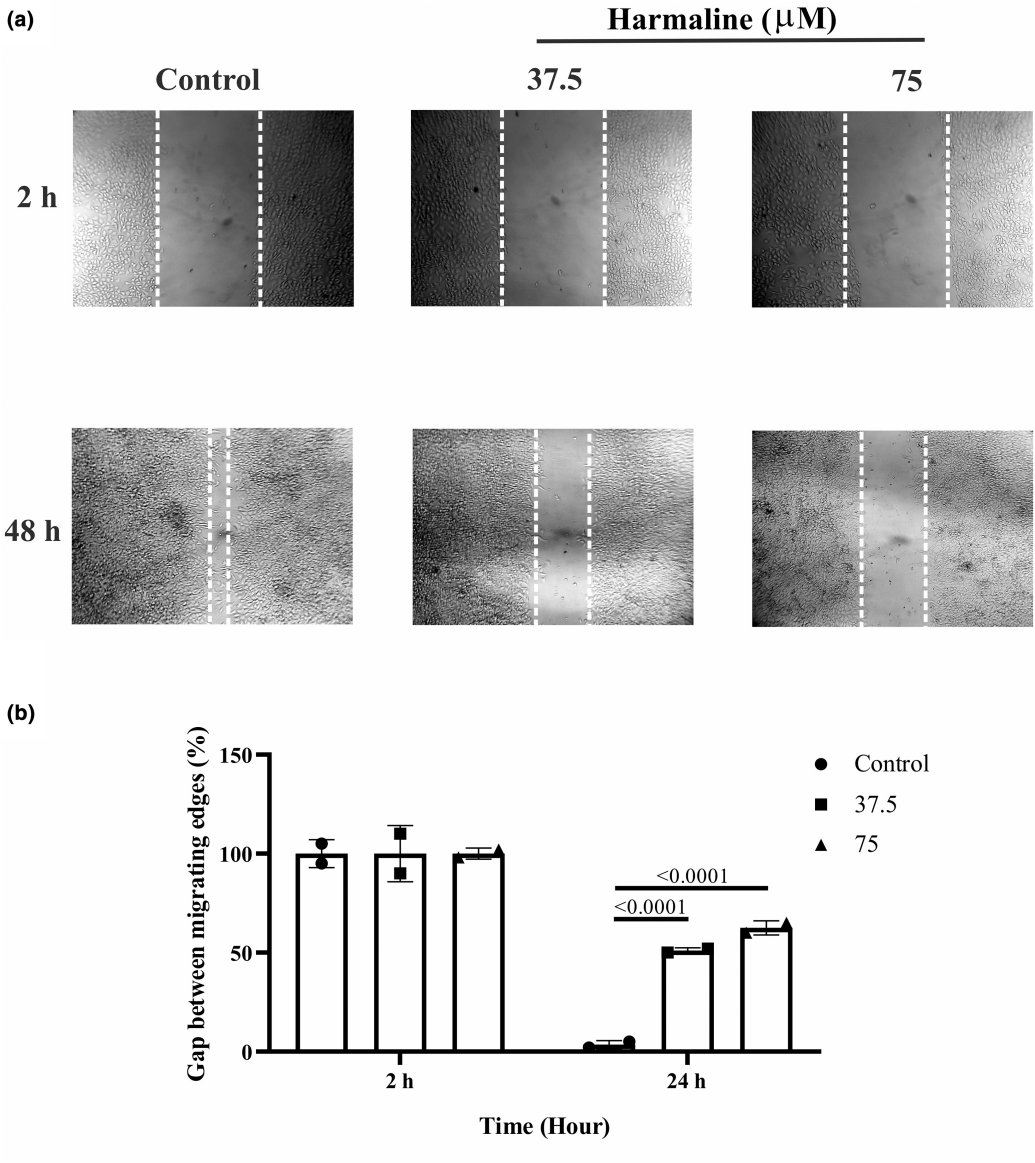
Effect of harmaline on the migratory capacity of A2780 cells in vitro. The migratory ability of harmaline‐treated A2780 cell line cells was analyzed using a wound‐healing assay. (a) Migration was monitored at 2 and 48 h. (b) The migration capacity of A2780 cells was significantly impaired by harmaline (37.5 and 75 μM) treatment compared to untreated control. The individual data points and significant *p*‐values are shown in each column, and the data are represented as mean ± SD.

**FIGURE 6:**
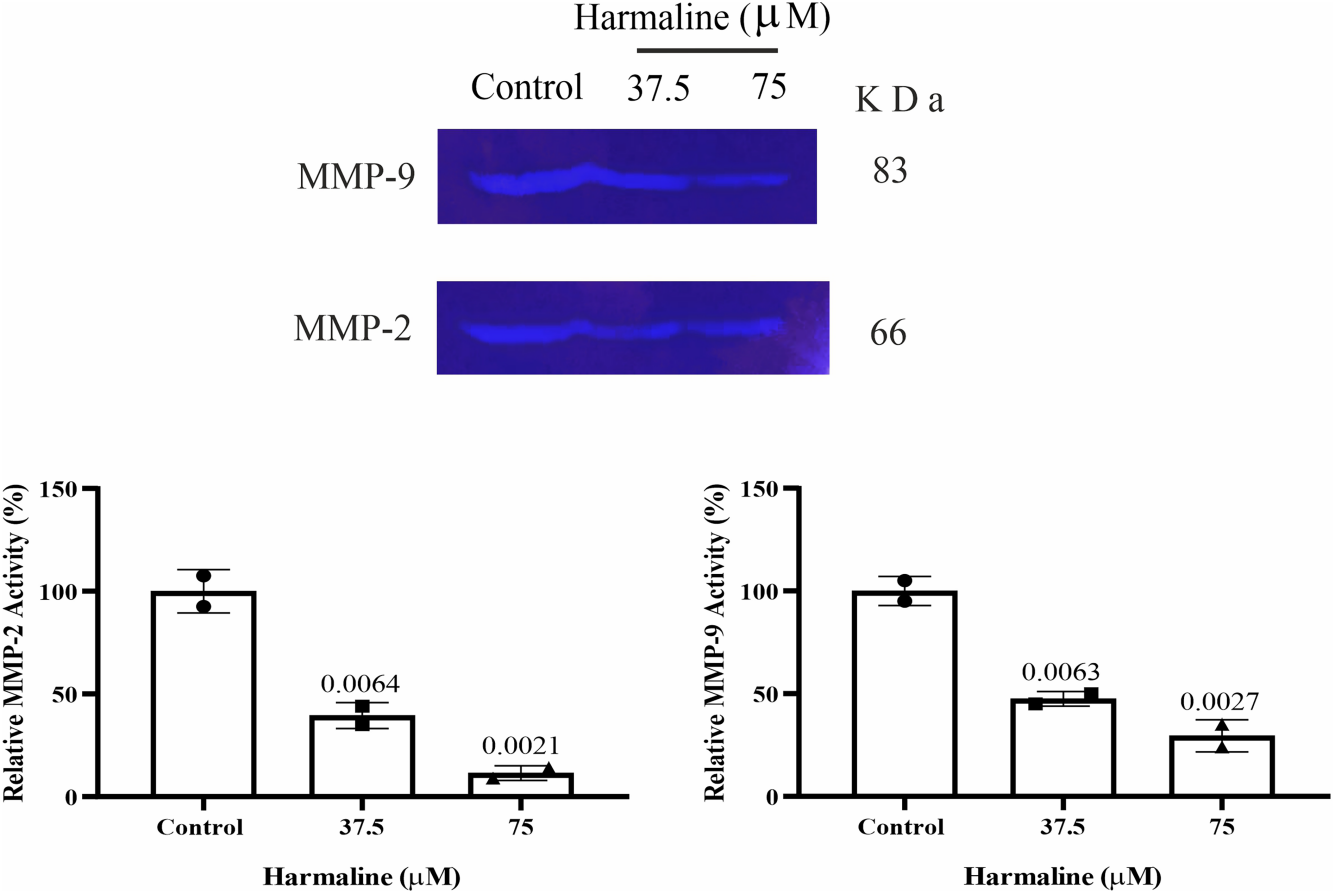
Harmaline decreased the MMP‐9 and MMP‐2 secretions of A2780 cells in vitro. The zymography test was performed to evaluate MMP‐2 and ‐9 activity. The bands represent the digested areas of gelatin by MMP‐9 and MMP‐2 after 24 h harmaline treatment (37.5 and 75 μM). The results showed that relative enzyme activities as a percent of the blank group for MMP‐2 and MMP‐9 significantly decreased after the treatment of A2780 cells with harmaline. Data presented as relative MMP‐9 and MMP‐2 activity versus untreated cells. The individual data points and significant *p*‐values are shown in each column, and the data are represented as mean ± SD.

Harmaline, at concentrations of 37.5 and 75 μM, was shown to inhibit the capacity of A2780 cells to migrate in comparison to an untreated control group (Figure 5a). Migration was monitored at 2 and 48 h. As shown in Figure 6b, harmaline was able to significantly decrease the capacity of A2780 cells to migrate in a dose‐dependent manner.

### 3.5 Harmaline attenuated the gelatinase activity of MMP‐2 and ‐9 in A2780 cells

A gelatin zymography technique was used in order to investigate whether harmaline, at concentrations of 37.5 and 75 μM, is able to mitigate the activity of MMP‐2 and MMP‐9. The bands with a molecular weight of 83, and 66 kDa represent active MMP‐9, and active MMP‐2, respectively. As compared to the control group, the enzymatic activity of the active forms of MMP‐2 and MMP‐9 in A2780 cells that were treated with 37.5 and 75 μM of harmaline for 48 h was decreased. As indicated in Figure 6, harmaline has a greater inhibitory impact on the activity of MMP‐2 than it does on MMP‐9. In addition, we evaluated the mRNA expression of MMP‐2 and MMP‐9, in order to further evaluate the antitumor activity of harmaline. As shown in Figure 3, the transcription levels of MMP‐2, and ‐9 was shown to be significantly lower at 35.5 and 75 μM of harmaline compared to the level found at untreated control.

### 3.6 Harmaline reduce invasion of A2780 cells

**FIGURE 7:**
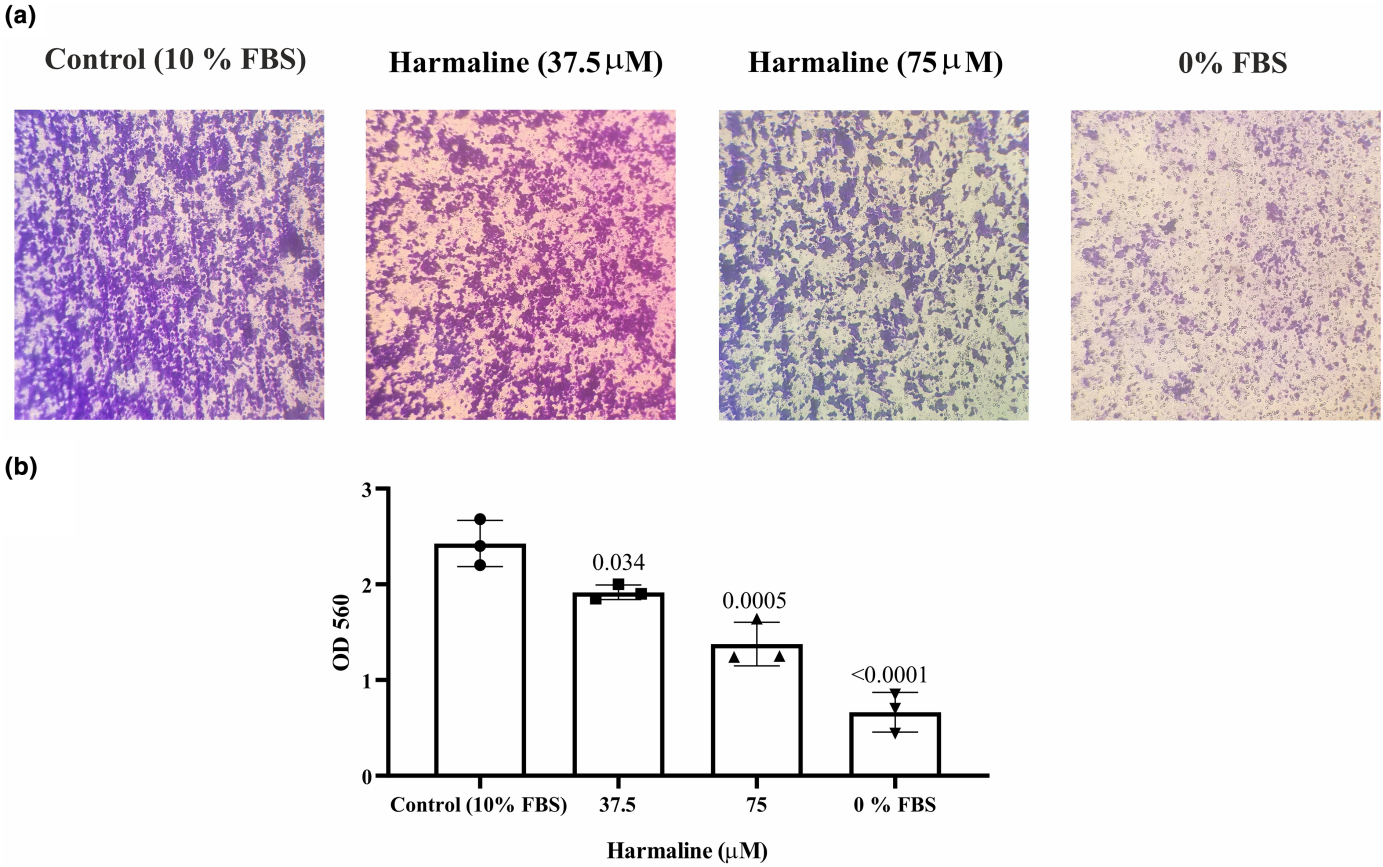
Harmaline inhibits the invasion of A2780 cells in vitro. (a) The impact of harmaline on the invasion of A2780 ovarian cancer cells was evaluated by treating the cells with 37.5 and 75 μM of harmaline for 24 h in media containing serum. (b) The invasive cells were then assessed using the cell invasion assay QCM™ 24‐Well Colorimetric Cell Migration Assay. The results showed that the number of invasive cells was significantly lower after treatment with harmaline compared to the control group, with values expressed as means ± standard deviation. The individual data points and significant *p*‐values are shown in each column.

It is possible that harmaline prevents A2780 cells from migrating, leading to a decrease in cell invasion. Therefore, the Matrigel‐coated transwell assay was used to assess the impact of harmaline on the invasion of ovarian cancer cells. The results in Figure 7a demonstrate that the number of cells that invaded after being treated with harmaline (at concentrations of 37.5 and 75 μM) for 24 h was significantly lower compared to the control group, and this reduction was dependent on the concentration of harmaline, indicating that harmaline effectively reduces the invasion of A2780 ovarian cancer cells in a concentration‐dependent manner (Figure 7b).

### 3.7 Toxicity studies

According to ProTox‐II, harmaline has been categorized as toxicity class‐4, which means that it is considered to have low toxicity. The predicted LD50 for harmaline was 550 mg/kg. The LD50 (median lethal dose) is a measure of the toxicity of a substance and is defined as the dose required to cause the death of 50% of the test population (usually laboratory animals) within a specified time period. The LD50 value is typically expressed in terms of the amount of substance per unit of body weight. It is a commonly used measure of acute toxicity in toxicology studies and is used to compare the relative toxicity of different chemicals. The results also showed that harmaline has no hepatotoxicity, carcinogenicity, immunotoxicity, mutagenicity, and cytotoxicity with a probability of 0.8, 0.61, 0.53, 0.85, and 0.72, respectively.

## 4 DISCUSSION

OC often remains undetected until it has spread beyond the ovaries (Arora et al., 2022). According to the current in vitro study, harmaline has dose‐dependent proliferative inhibition effect on A2780 cells. Furthermore, our findings showed that harmaline is safe and has minimal effects on NIH/3T3 normal cells at doses that reduced the viability of A2780 cells. NIH/3T3 cells is a widely used embryonic fibroblast cell line in early‐stage drug discovery to test the efficacy and safety of potential drugs (Rahimi et al., 2022).

The results of the study suggest that harmaline attenuated the growth of A2780 cells by inducing apoptosis and cell arrest. Moreover, the study indicates that harmaline has influenced the transcription levels of Bax and p53 genes, which are responsible for apoptosis regulation. Additionally, harmaline has caused an overproduction of ROS. This overproduction of ROS could have contributed to the antitumor effects of harmaline on A2780 cells.

The MTT analysis revealed that treatment with harmaline led to a significant dose‐dependent reduction in cell viability in A2780 cells. This indicates that harmaline is cytotoxic to A2780 cells. In addition, harmaline has cytotoxic effects in other cancer cell lines, such as human prostate cancer cells (IC50: 8–10 μM), non‐small‐cell lung cancer cells H1299 (IC50: 48.16 ± 1.76 μM), breast cancer cells 4T1 (IC50: 144.21 μM), and A549 cells (IC50 = 67.9 ± 2.91 μM) (Rashidi et al., 2022; Roy et al., 2020). The cytotoxic effect of harmaline may be due to its ability to activate apoptosis or cell death in cancer cells. Wang et al. indicated that harmaline (IC50 < 4 μM) was able to significantly suppress the proliferation of gastric tumor cells suggesting harmaline was able to achieve this effect at relatively low concentrations. According to this research, harmaline has the potential as a natural anticancer agent, but its efficacy varies depending on the type of cancer and cells used (Wang et al., 2015).

NIH/3T3 cell line is less sensitive to harmaline than the A2780 cell line, which is a positive finding as it indicates that harmaline has selective toxicity toward cancer cells rather than normal cells. Consistent with this finding, harmaline was reported to have no obvious cytotoxic effect in mouse fibroblast cells (NIH/3T3) (Wang et al., 2015).

The control of the apoptosis is critical in tumor development and propagation (Gousias et al., 2022). Any disruption or perturbation in apoptotic pathways can lead to uncontrolled proliferation and resistance to chemotherapy of tumor cells. Our results showed that enforce of apoptosis is one of the mechanisms by which harmaline can reduce the growth of A2780 cells. It has been shown that harmaline induces apoptosis in cancer cells by activating the caspase cascade, which is a key pathway in apoptosis. It has been reported that harmaline induces an apoptotic pathway in SGC‐7901 human gastric cancer cells, HeLa, breast cancer cells, and liver cancer cells (Hashemi Sheikh Shabani et al., 2015; Wang et al., 2015; Xu et al., 2018).

Gene expression of p53 and Bax were upregulated in harmaline‐treated A2780 cells. p53 is a tumor suppressor gene that transcriptionally regulates multiple genes in different pathways and plays a momentous role in regulating cell division and preventing the formation of cancerous cells (Hernández Borrero & El‐Deiry, 2021). p53 activates Bax, which is a proapoptotic protein that can trigger programmed cell death in response to agents which trigger p53‐mediated A2780 cell death.

Increased intracellular ROS participate in inducing apoptosis (Redza‐Dutordoir & Averill‐Bates, 2016). ROS generates by cells during normal metabolic processes. However, excessive ROS production can produce adverse effects on cellular components including proteins, lipids, and nucleic acids, which results in the induction of cell death signaling pathways (Li et al., 2018; Siddiqui et al., 2017). Harmaline increases the production of ROS in A2780 cells, which, in turn, can trigger apoptosis. In line with us, shreds of evidence supported that harmaline induces the generation of intracellular ROS. For instance, harmaline was found to exert a potent cytotoxic effect by increasing the generation of ROS in HeLa cervical cancer cell line (Bhattacharjee et al., 2018). In addition, the activation of p53 by ROS‐induced DNA damage can lead to the transcription of Bax and other genes that play a critical role in triggering apoptosis, which is an important mechanism of activation of apoptosis in harmaline‐treated cells (He & Simon, 2013).

MMP‐2 and MMP‐9 are known to be participated in the process of cell invasion (Jalili‐Nik et al., 2019), so the downregulation of these genes may result in a reduction in the ability of A2780 cells to invade surrounding tissue. The result of gene expression revealed that harmaline could reduce the invasiveness of A2780 cells by downregulating MMP‐2 and MMP‐9 genes. To test the anti‐migration activity of harmaline in vitro, we evaluated cell motility in response to harmaline by wound assay. The wound assay is a common in vitro method used to evaluate cell migration and invasion. It involves creating a wound or scratch in a cell monolayer and then monitoring the ability of the cells to migrate and close the wound overtime (Cory, 2011). Our study showed that harmaline was able to inhibit the motility of A2780 cells in a dose and time‐dependent manner. The measurement of cell migration in these clusters may be complicated by the fact that cell proliferation can also contribute to the overall movement of the cluster. As cells divide and replicate, they can push other cells forward, creating the appearance of migration even if individual cells are not actively moving (Moffitt et al., 2019). To accurately measure cell migration in these types of clusters, it may be necessary to account for cell proliferation and distinguish between true migration and movement caused by cell division. This can be done through techniques such as live‐cell imaging, which allows researchers to track individual cells overtime and observe their movement patterns (Rodriguez et al., 2005). However, the wound assay has provided valuable insights into the effects of harmaline on cell migration. Further investigation using live imaging is needed to fully understand the mechanism of harmaline on cell migration.

Zymography showed a clear reduction in gelatinolytic activities of MMP‐2 and MMP‐9 in A2780 cells exposed to harmaline versus to the untreated group. This suggests that harmaline may have an inhibitory effect on the activity of MMP‐2 and MMP‐9, which could potentially reduce the ability of A2780 cells to invade surrounding tissues and disseminate.

MMPs play a main role in tumor invasion and migration (Jalili‐Nik et al., 2019; Kumar et al., 2018), and their suppression by harmaline had a considerable negative impact on cell motility and invasion. Evidence has indicated that MMP‐2 and ‐9 are upregulated in OC and both MMPs play key roles in the migration of tumor cells (Afzal et al., 1998). In line with our finding, Hamsa et al. reported that harmine, a pharmacological β‐carboline alkaloid found in *P. harmala* L., decreased the levels of MMP‐2 and MMP‐9. They also indicated that harmine dose dependently reduces the ability of HUVEC cells to move toward the wound area (Hamsa & Kuttan, 2010). These results demonstrated that through decreasing the expression of MMP‐2 and ‐9, harmaline prevented cancer cells from completing the essential steps of metastasis, ultimately leading to the suppression of this process.

The current study has some limitations. First, we investigated the antitumor activities of harmaline in ovarian cell line A2780 which is characterized by its ability to invade surrounding tissues as a key feature of cancer metastasis. However, A2780 cells are derived from ovarian epithelial cells, which are the most common type of cells that give rise to OC. But this cell line may not be representative of other types of OC cells. Second, it is also worth noting that in vitro studies using cell lines have limitations and may not accurately reflect the in vivo effects of treatment. In addition, it should be noted that the impact shown by harmaline in the current study is in the high micromolar range which may undermine its potential as a therapeutic compound.

## 5 CONCLUSION

The findings of the study indicate that harmaline could decrease proliferation and reduce metastatic behavior A2780 OC cells through induction of apoptosis and inhibition of invasion and migration. Additionally, harmaline has caused an overproduction of ROS. This overproduction of ROS could have contributed to the anticancer effects of harmaline against OC cells. Further studies using animal models or clinical trials are urgent to confirm the potential therapeutic benefits of harmaline in cancer therapy.

## AUTHOR CONTRIBUTIONS

**AB**: Methodology, conceptualization, formal analysis, validation, funding acquisition, and writing—original draft. **SASR**: Formal analysis, validation, and writing—review and editing. **MTK**: Methodology, formal analysis, and validation. **RJ**: Resources, supervision, and writing—review and editing. **AS**: Methodology, formal analysis, and validation. **GF**: Supervision and review and editing. All Authors read and confirmed the manuscript.

## FUNDING INFORMATION

This study supported by Mashhad University of Medical Sciences, Mashhad, Iran (code: 4011304).

## CONFLICT OF INTEREST STATEMENT

The authors declare that there is no conflict of interest.

## ETHICS STATEMENT

The study was approved by Ethics Committee of Mashhad University of Medical Sciencesو, Mashhad, Iran (IR.MUMS.IRH.REC.1401.016).

## Data Availability

The data supporting findings of this study are available on request from the corresponding author.
